# Optical conductivity of a Bi_2_Se_3_ topological insulator with a THz transparent top gate

**DOI:** 10.1515/nanoph-2023-0690

**Published:** 2024-02-05

**Authors:** Craig S. Knox, Matthew T. Vaughan, Nathan R. Fox, Ahmet Yagmur, Satoshi Sasaki, John E. Cunningham, Edmund H. Linfield, Alexander G. Davies, Joshua R. Freeman

**Affiliations:** School of Electronic and Electrical Engineering, University of Leeds, Leeds LS2 9JT, UK; School of Physics and Astronomy, University of Leeds, Leeds LS2 9JT, UK

**Keywords:** topological insulators, THz time domain spectroscopy, quantum transport

## Abstract

We have performed an investigation into the optical conductivity and magnetotransport properties of top-gated devices patterned on the topological insulator Bi_2_Se_3_ in order to determine the relative effects of the different carrier species that exist within these novel materials. We find that the topologically protected surfaces within our samples are partially screened from the action of the gate by trivial band-bending states formed at the top surface of the topological insulator. Despite this, the mobility of the topological surface carriers is significantly affected by the application of an external gate bias. Additionally, we find that the optical conductivity response is dominated by the topologically protected surface states, and that the optical conductivity is particularly sensitive to the scattering caused by the topological surfaces coupling to trivial states, arising from the bulk or band-bending induced surface states. These results will have interesting applications to the design of future plasmonic devices that incorporate topological materials.

## 1 Introduction

Topological insulators (TIs) are interesting novel materials that host Dirac-like surface-states surrounding an insulating bulk [1], [2]. These states are spin-momentum locked, so that a 180° backscattering requires a spin-flip. This results in transport that is coherent over much longer distances than conventional materials, due to elastic backscattering being forbidden. These properties have inspired a great deal of interest in these material systems, particularly in the field of plasmonics, where the protection against backscattering should give rise to high-mobility, long range plasmons [3], [4]. Additionally, the spin momentum locking that gives rise to that protection should allow the observation of pure spin-plasmons, where only spin information is transmitted [5].

However, in many TI material systems, the bulk is not insulating but instead can dominate the electronic properties of the system [6], [7]. This complicates analysis of the electronic properties of the TI material, as the response of the bulk and surface are hard to separate. This is especially prominent in optical analysis of TI materials, where the response of the bulk and surface carriers can be hard to disentangle [3], [8], [9]. Notwithstanding this, the optical conductivity of these TI materials remains a useful tool to characterise these protected surface states, in part due to the sensitivity of the optical conductivity to scattering events such as interband transitions [10], [11], [12].

By applying an electric field to a device, for example from an electrostatic gate, the confining potential of a system can be altered, which will alter the electrical properties of the system. This is especially useful in a TI system, as the gate should only effect the surface closest to the gate electrode. This should allow the effect of one set of topological surface states on the electrical and optical properties to be distinguished from the topologically non-trivial bulk carriers and the topologically protected carriers on the opposite surface. However, due to their 2D nature, a gate bias applied to one surface will not be fully screened from the bulk or the opposite surface [13], which complicates analysis. Additionally, the topologically protected surface states can co-exist alongside trivial surface states formed by band bending [6] or finite-size effects [14]. Therefore, in order to fabricate optoelectronic devices that take full advantage of these topological surface states, we need to establish to what extent the topological surface states contribute to the optical properties of the material system, and how external electric fields modulate those optical properties.

Electrostatic gates have been used on TI materials to investigate and control the low frequency electronic properties of the device [6], [15], [16] and how the different carrier species evolve under an applied electric field. So far, ionic fluids have been used to apply an electric field to a TI in order to modulate their optical properties in the THz regime; either by charge transfer [17] or by ionic fluid gating [18], [19]. While these techniques allow incredibly large electric fields to be applied to TIs, it is difficult to modulate the applied field, especially at cryogenic temperatures, as this would require the re-orientation of ions within the fluid. Additionally, very few studies have coupled the THz analysis to high-field magnetotransport studies in order to distinguish the effects of bulk and topologically protected carriers on the optical properties.

Here, we show a study of the effects of a top gate bias, applied from a THz-transparent metallic top-gate on a Bi_2_Se_3_ TI thin-film via both low-frequency magnetotransport measurements and THz studies of the optical conductivity. Using these complementary techniques allows us to determine that the optical properties of the TI sample are dominated by the topologically protected surface states. Additionally, we find that the surface states within our samples are screened from the action of a top gate by band-bending states formed on the surface of the TI, and that the coupling between the topologically trivial and non-trivial surface states can be probed by the change in the optical properties as a function of applied top gate bias.

## 2 Materials and methods

The 17.8 nm thick Bi_2_Se_3_ sample was prepared within a solid-source MBE system, with a base pressure of $\approx 1\times 10^{-10}\;$ Torr. The sample was grown on c-plane oriented Al_2_O_3_ substrates by co-deposition of evaporated bismuth and selenium from dual-filament Knudsen cells. The sample was grown at a substrate temperature of 235 °C, measured by a thermocouple attached to the sample manipulator, before cooling under a selenium flux at a rate of 3 °C/min, in order to improve the quality of the surface of the TI material.

**Figure 1:**
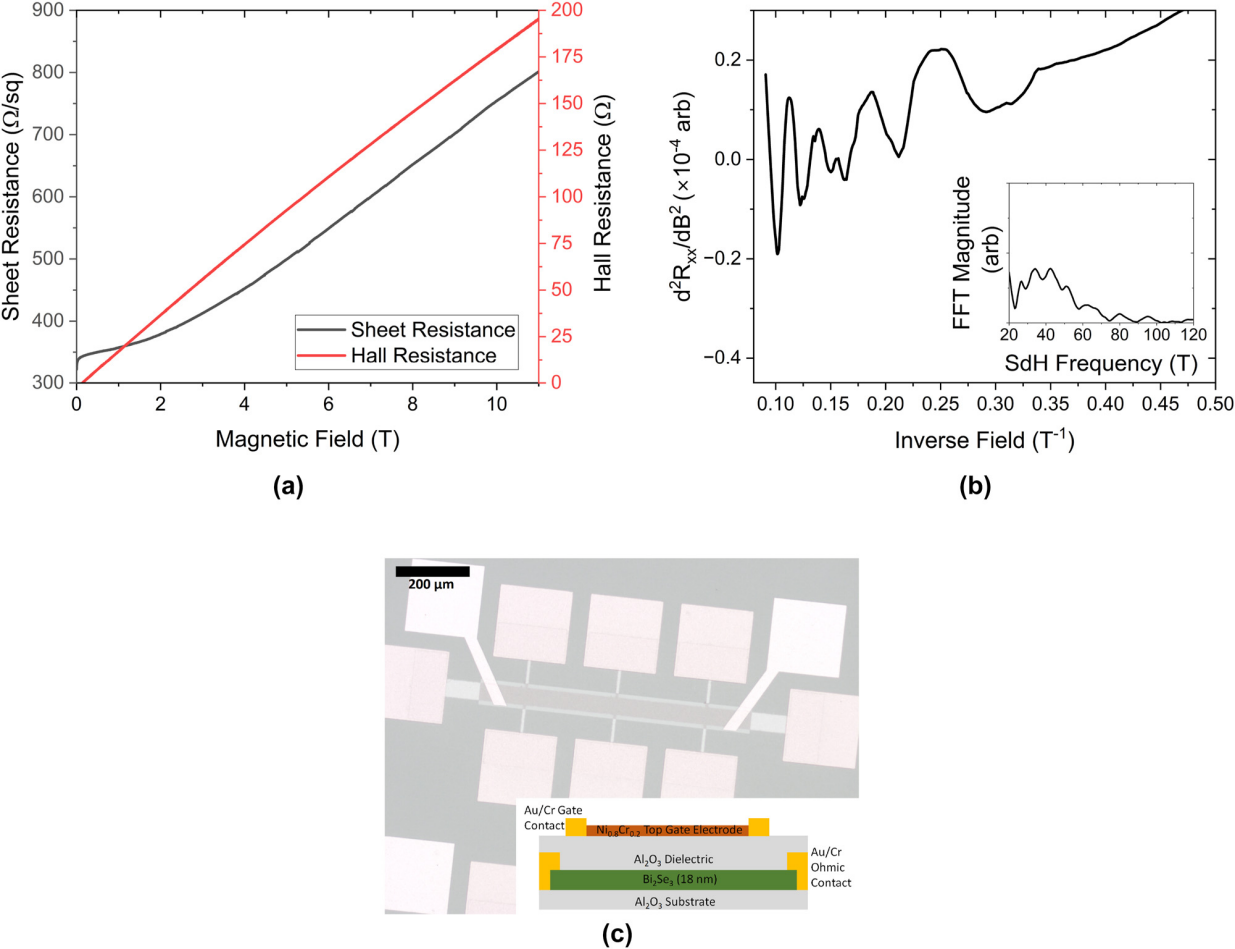
Low-temperature magnetotransport characterisation of the Bi_2_Se_3_ sample at 0.4 K. (a) Shows the sheet (longitudinal, black) and Hall (red) resistances for the sample up to 11 T. The dip in sheet resistance at 0 T is characteristic of the weak anti-localisation displayed by this material. Note that the Hall trace is non-linear as a function of field, indicating the presence of two carrier gasses with differing mobilities. (b) Shows the second derivative of the sheet resistance in (a) as a function of 1/*B*, revealing periodic SdH oscillations. The inset shows the FFT of (b), revealing a 42 ± 1 periodicity to the oscillations. (c) Shows an optical micrograph of a gated Hall bar used in this study. The orange squares are Cr/Au ohmic contacts under the Al_2_O_3_ dielectric, the yellow-white squares are Cr/Au gate contacts and the brown square is the thin Ni_0.8_Cr_0.2_ top gate electrode on top of the Bi_2_Se_3_ channel. The inset shows a schematic side-view of the device structure.

The crystallographic properties of Bi_2_Se_3_ samples were subsequently analysed by X-ray reflectivity and X-ray diffraction, using Cu K_α_ radiation, before being diced for fabrication. The sample was patterned into Hall bars and 1 mm^2^ slabs for substrate referenced THz-time domain spectroscopy (TDS) by chemical wet etching. The 50 nm thick Cr/Au ohmic contacts are subsequently thermally evaporated and lifted-off before a 1 nm thick layer of Al is sputtered across the entire sample and left to oxidise for at least 2 h, forming a layer of Al_2_O_3_, before a further 25 nm thick layer of Al_2_O_3_ is deposited by atomic layer deposition. Without the sputtered Al_2_O_3_ seed layer, the chemically passive surface of the Bi_2_Se_3_ prevents the adhesion of the trimethylaluminium atomic layer deposition precursor and no dielectric is formed. A 5 nm thick layer of Ni_0.8_Cr_0.2_ is then subsequently thermally evaporated as a top gate electrode, with 50 nm thick Cr/Au gate contacts evaporated as a final step. An optical micrograph of a Hall bar used in this study is shown in the inset of Figure 1. It is worth noting that deposition of the Al_2_O_3_ dielectric results in additional carriers being visible in the THz-TDS measurements. Further details can be found in the Supplementary Information [20].

Transport measurements were performed in a modified Oxford Instruments dilution refrigerator [21] at a temperature of 300 mK, with a maximum field of 11 T. Transverse and Hall resistances were measured with standard locking techniques with a source-drain bias current of 100 nA, at a frequency of 126 Hz. In order to perform the gate-dependent magnetotransport measurements, the magnet was swept between 0 and 11 T at a rate of 0.1 T/min while holding the gate bias constant.

The THz-TDS measurements were undertaken at 4.2 K in a continuous flow He cryostat. The sample to be measured, along with a bare sapphire reference, was mounted on the cold finger of the cryostat, which was subsequently evacuated and cooled to the required temperature. The time-domain signal was then acquired using 20 fs pulses from a Ti–sapphire laser, a delay stage and two LT-GaAs-on quartz photo-conductive antennas, with a spectral range of 0.5–4.5 THz. Further details can be found in Ref. [22]. A top gate bias was applied between the transparent NiCr [23] top gate-electrode and the TI and kept constant throughout each THz-TDS scan.

The time-domain data are then trimmed (with a boxcar window) to remove unwanted Fabry–Perot reflections and zero-padded by 5 ps in either direction before a Fourier transform was taken. The complex transmittance of the topological insulator is then defined as $T\left(\omega \right)=\frac{E_{\text{sample}}\left(\omega \right)}{E_{\text{reference}}\left(\omega \right)}$ where *E*(*ω*) is the complex result of the Fourier transform. From the complex transmittance, the optical conductivity can then be determined via [24];

(1) $$\tilde{\sigma }\left(\omega \right)=\left(\frac{1}{T\left(\omega \right)}-1\right)\frac{1+n_{\text{reference}}}{Z_{0}d}$$

where *Z*_0_ is the impedance of free space, *d* is the thickness of the topological insulator film and *n*_reference_ is the refractive index of the substrate, determined from the sapphire reference.

## 3 Results

### 3.1 Low temperature magnetotransport

The resistance of the sample decreases with decreasing temperature, indicative of phonon-freeze out. This is typical of samples where the topologically trivial, bulk carriers dominate the transport as otherwise the resistance increases with decreasing temperature as residual carriers freeze out [25]. The as-cooled (before the application of any gate bias) low-temperature magnetotransport of the Bi_2_Se_3_ sample is shown in Figure 1. There is a strong non-linearity in the Hall resistance as a function of applied magnetic field, indicative of multiple carrier species with differing mobilities [26], [27]. Fitting the non-linear Hall trace to a two-band model [27] reveals the presence of a high carrier density ((3.5 ± 0.2) × 10^13^ cm^−2^), low mobility (380 ± 20 cm^2^ V^−1^ s^−1^) band and a low carrier density ((2.4 ± 0.1) × 10^12^ cm^−2^), high mobility (1120 ± 50 cm^2^ V^−1^ s^−1^) band. Due to limitations of the model, more than two carrier species cannot be accurately distinguished, so we have restricted our fitting to two carrier types, which we assign to the topologically trivial (high carrier density, low mobility) and topologically non-trivial carriers (low carrier density, high mobility).

We then take the second derivative of the sheet resistance to remove the smoothly varying background and find oscillations that are periodic in 1/*B*. These Shubnikov de-Haas (SdH) oscillations (shown in 1b) are characteristic of transport of highly mobile carriers undergoing cyclotron motion and oscillate with a frequency proportional to their carrier density. Taking the FFT of these oscillations (shown in the inset of 1b) reveals a broad frequency spectrum, centred at 42 ± 1 T. This, along with the envelope function of the oscillations that appears to show a beating pattern, reveals at least two sets of oscillations, one which is onset from a field of 4 T (0.25 T^−1^ in Figure 1(b)) and another higher frequency set of oscillations, which is onset from a field of 6 T (0.17 T^−1^ in Figure 1(b)), superimposed on top of the lower frequency oscillations. This implies the coexistence of at least two carrier species within the TI sample. It’s worth noting that the frequency revealed by the FFT ((2.03 ± 0.05) × 10^12^ cm^−2^) corresponds to carrier densities, which are not comparable to that of the high-carrier density, low mobility band extracted from the non-linear Hall trace but which are comparable to the carrier density of the high mobility surface states.

**Figure 2:**
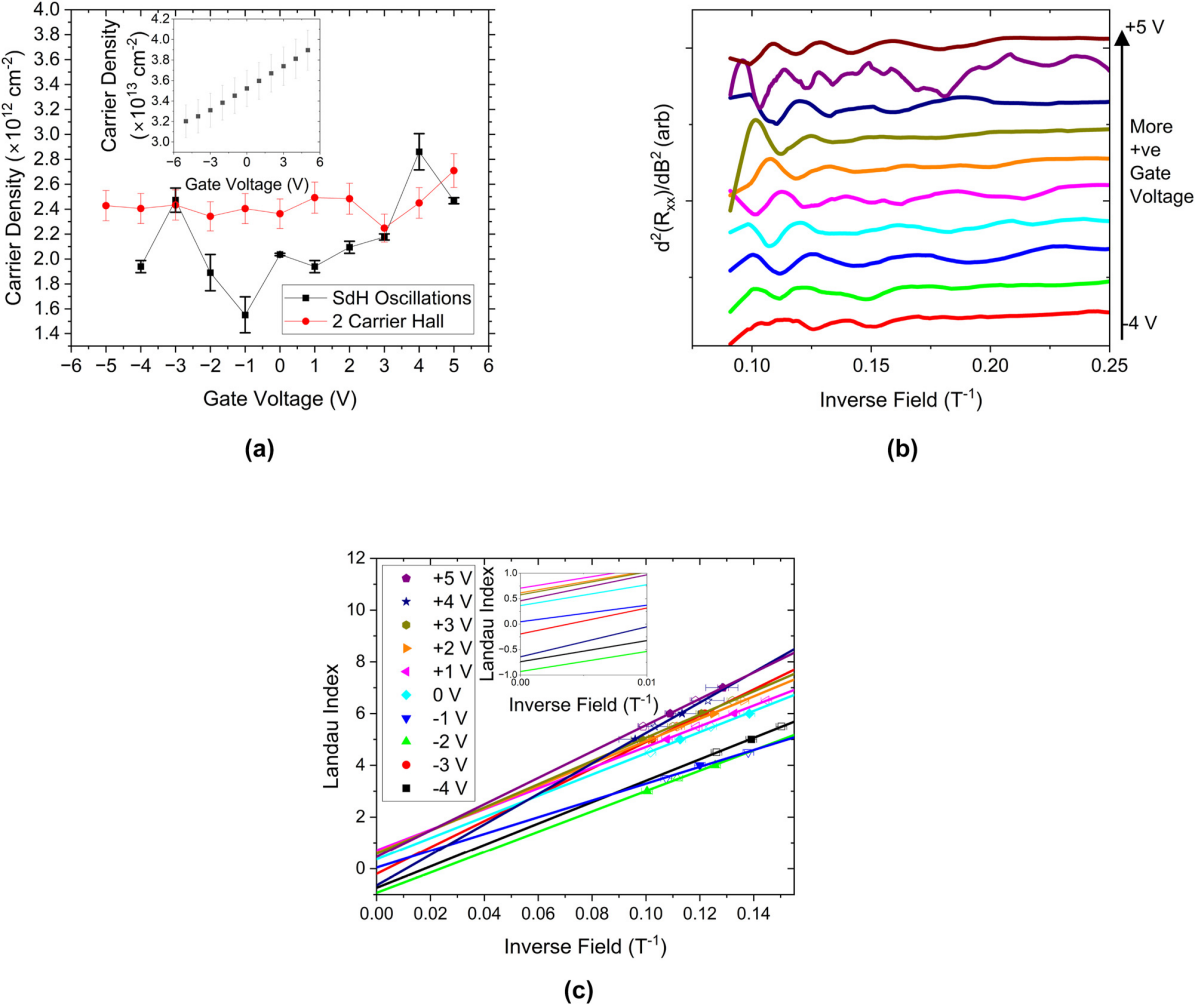
Gate-dependent measurements of the magnetotransport in the Bi_2_Se_3_ sample. (a) Shows the variation of the carrier densities extracted from the high mobility, low carrier density band from the two carrier Hall fitting and the most prominent SdH oscillations. The inset shows the variation in the carrier density of low mobility band as a function of gate bias. (b) Shows the second derivative of the sheet resistance as a function of gate between ± 5 V as a function of inverse field, highlighting the periodic SdH oscillations. Curves are artificially offset for clarity. (c) Shows a Landau fan of the low frequency, more prominent SdH oscillations, where closed symbols are SdH minima (maxima in the second derivative) representing full, spin degenerate, Landau levels, and open symbols are SdH maxima (minima in the second derivative) representing half-full Landau levels. The inset shows the *y*-intercepts of the linear fits to the data, revealing that all fits to oscillations with positive biases have an intercept of 0.5, indicative of topologically interesting transport, whereas the same fits to the data taken with negative biases have an intercept of 0 or ±1, indicative of topologically trivial transport.

The magnetotransport measurements were then repeated applying a top gate bias between ±5 V. When we consider the two-band Hall fitting, as shown in Figure 2(a), it becomes apparent that only the carrier density of the topologically trivial carriers are significantly affected by an applied gate bias. This is supported by measurements of the SdH oscillations (shown in Figure 2(b)), where the position and frequency of the more prominent, low frequency oscillations is not significantly altered by an applied gate bias. This is somewhat counter-intuitive, as one would expect the gate to modulate the carriers on the top surface over the bulk due to their physical proximity. While the carrier density of these oscillations is largely robust against an applied gate bias, it is clear to see that the field at which these oscillations become apparent is dependent on an applied gate bias, with more positive biases showing oscillations at lower fields (higher inverse fields). This implies that, while the gate bias does not significantly change the carrier density of the high mobility carriers, the mobility of those carriers is affected by a gate bias.

In order to determine the origin of the SdH oscillations, we construct a Landau fan of the most prominent, low frequency SdH oscillations, shown in Figure 2(c). Oscillations arising from a Dirac cone, such as those that give rise to the topologically protected surface states, have an additional π phase-shift when compared to their conventional counterparts [28]. As such, the *y*-intercept of a Landau fan will be ±0.5 for topologically protected carriers, and ±1 or 0 for topologically trivial carriers. Interestingly, when a positive, or 0 V, gate bias is applied, the *y*-intercept is ± 0.5, but negative gate biases results in a trivial intercept.

It is worth noting that forming a Landau fan out of the less prominent, higher frequency oscillations invariably reveals a trivial intercept. Due to this trivial phase, the oscillations do not arise due to a TI surface, and the fact these oscillations are robust against gate voltage rules out band bending states at the top surface. Therefore, we assign the high-frequency oscillations to band-bending induced states at the substrate–TI interface.

### 3.2 Terahertz time domain spectroscopy

**Figure 3:**
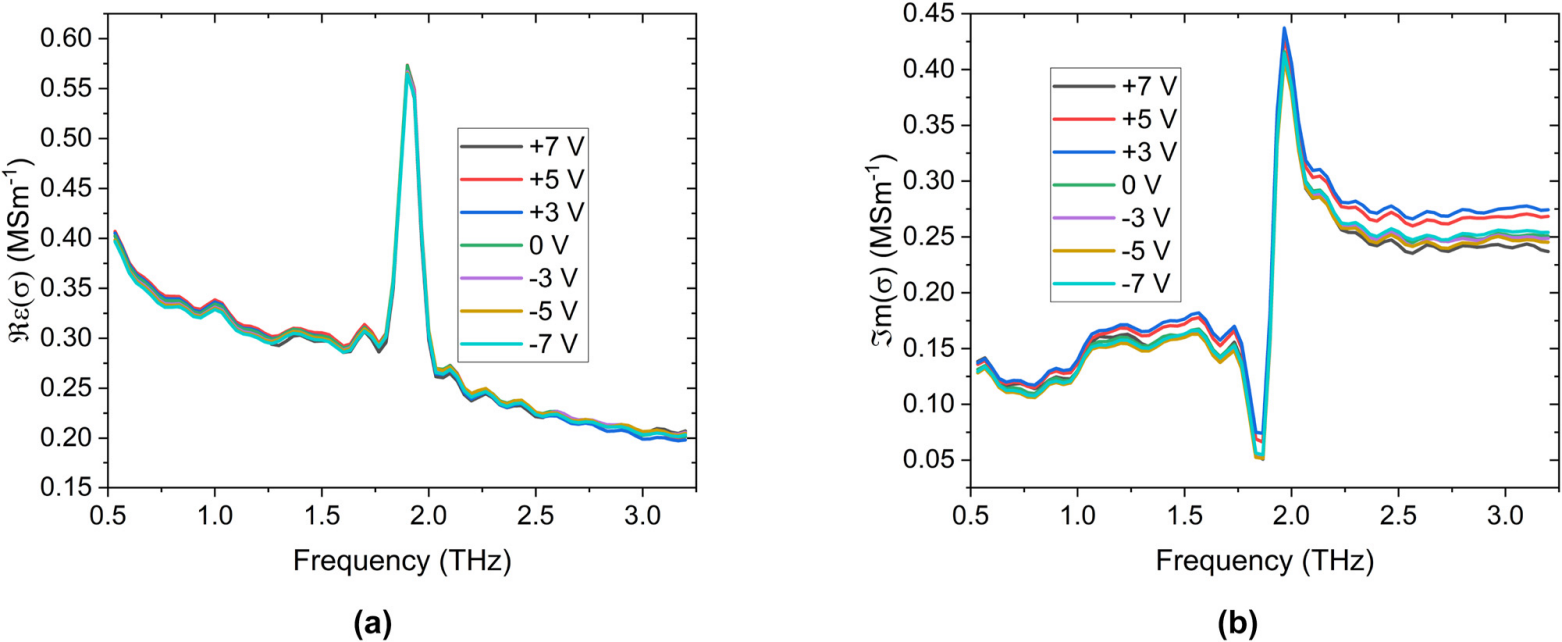
Optical conductivities of the Bi_2_Se_3_ sample as a function of top gate bias at 4.2 K. (a) Shows the real part of the conductivity, and (b) details the imaginary part. Note that the IR-active $E_{u}^{1}\;$ phonon does not change with applied gate bias, showing that the structural properties of the film are not altered by an applied gate bias.

The optical conductivity of the Bi_2_Se_3_ sample as a function of applied gate bias is shown in Figure 3. As with similar studies on the optical properties of Bi_2_Se_3_ samples [8], [12], [29], [30], [31], [32], the conductivity spectrum is composed of an extremely prominent $E_{u}^{1}\;$ phonon at 2 THz with a Lorentzian response and Drude-like background arising from free carriers. The optical conductivity can, therefore, be fit by equation (2), which contains a Drude term incorporating the DC conductivity *σ*_*DC*_ and Drude scattering time *τ*, a Lorentzian describing the optical response of the phonon, including the phonon central frequency $\Omega_{E_{u}^{1}}^{2}$ , phonon scattering time $\gamma_{E_{u}^{1}}$ and amplitude of the phonon’s optical response $A_{E_{u}^{1}}^{2}$ , and a final term to account for the background permittivity, *ϵ*_∞_.

(2) $$\tilde{\sigma }\left(\omega \right)=\frac{-\sigma_{DC}}{i\omega \tau -1}-\frac{i\epsilon_{0}\omega A_{E_{u}^{1}}^{2}}{\Omega_{E_{u}^{1}}^{2}-\omega^{2}-\left(i\omega \gamma_{E_{u}^{1}}\right)}-\left(\epsilon_{\infty }-1\right)i\omega \epsilon_{0}$$

**Figure 4:**
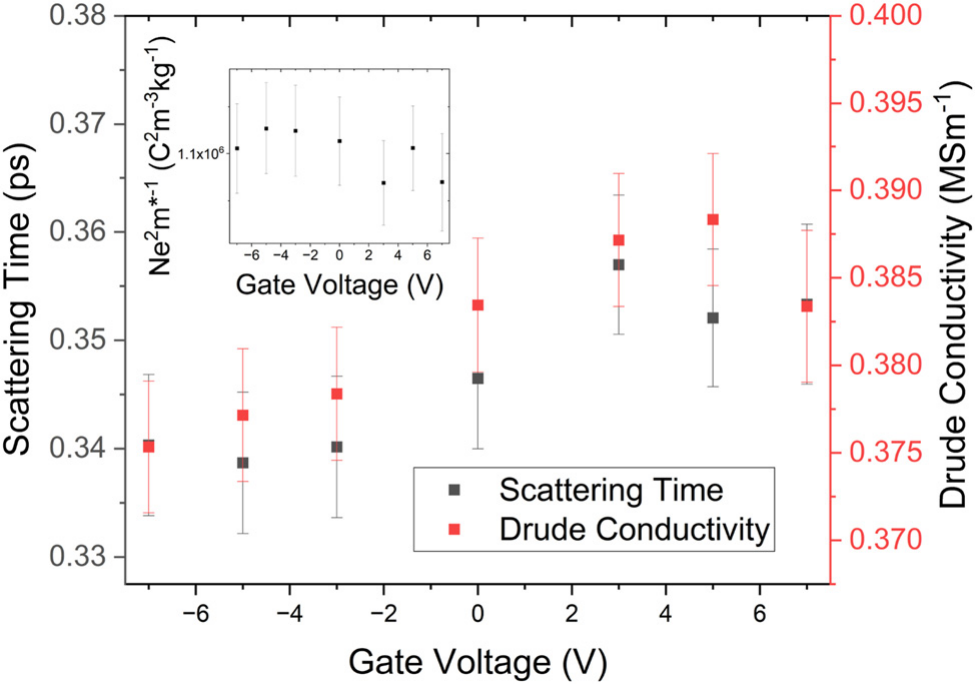
Results of fitting equation (2) to the data in Figure 3. Note how both the conductivity and scattering time change across 0 V gate bias. The inset highlights the constant nature of *σ*/*τ*, indicating that the change in the THz response arises from a change in the mobility of the carriers, rather than a change in carrier density.

As shown in Figure 3(a), the optical response of the phonon at 1.91 ± 0.01 THz is unaffected by the application of a gate bias. This is expected, as the gate should not be able to induce a structural transition within the TI sample. More interesting is the effect that the gate has on the Drude background. There is a small change in the slope of the Drude roll-off in the real conductivity and a significant change in the imaginary conductivity. By fitting this with the complex conductivity described in equation (2) (summarised in Figure 4), we find that the extracted and scattering time-scale changes around 0 V gate bias, changing from 0.354 ± 0.007 ps at positive biases to 0.340 ± 0.007 ps at negative biases. We also note that the trends in scattering time-scale and Drude conductivity are remarkably similar. As $\sigma_{DC}=\frac{ne^{2}\tau }{m^{*}}$ , where *n* is the carrier density (gate bias dependent) and m* is the effective mass (independent of gate bias), *σ*/*τ* should give an insight as to how the carrier density varies as a function of gate bias, shown in the inset of Figure 4. As *σ*/*τ* does not appear to vary significantly with gate bias, we can conclude that the carrier density probed by the THz radiation does not significantly vary, but the mobility of the carriers is changed by the applied bias.

**Figure 5:**
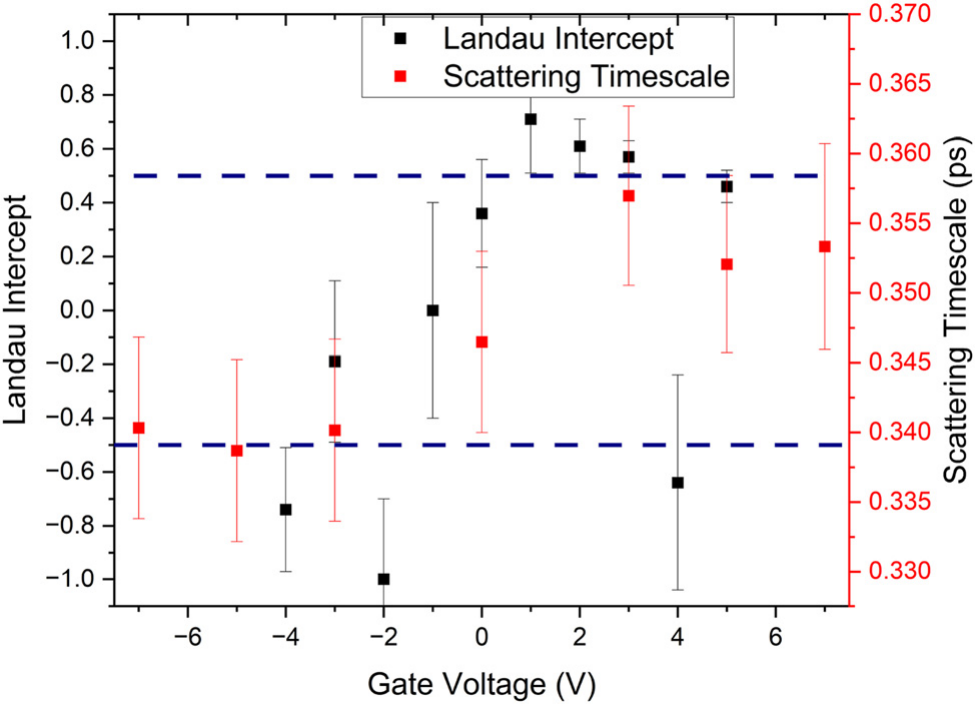
Comparison between the THz scattering time extracted from Figure 4 and the *y*-intercept of the Landau fan in Figure 2(c). The intercept of ±0.5, consistent with topologically non-trivial Dirac carriers, is highlighted with dashed blue lines. Note how the drop in THz scattering time is coincident with the loss in the π phase characteristic of topologically protected Dirac carriers.

These observations, taken in tandem, imply that the topologically protected surface carriers make a dominant contribution to the THz conductivity spectrum, and the bulk carriers are masked due to their low mobility [3], [8]. If the bulk carriers made significant contributions to the THz conductivity spectrum, we would expect to see the change in carrier concentration seen in the inset of Figure 2(a) mirrored in the inset of Figure 4, which is not the case. Secondly, due to the change in THz scattering time around 0 gate bias, we can infer that some new scattering mechanism becomes available as we apply a negative gate bias, which is coincident with the loss of the π phase characteristic of Dirac carriers, as shown in Figure 5. We attribute this to a low-mobility trivial carrier gas formed by the action of the top gate. From the inset of 2a, it is clear that only the topologically trivial carriers are modulated by the action of a gate bias applied to the Bi_2_Se_3_ sample, but the surface carriers are not modulated by the application of the bias, despite being physically closer to the gate electrode than the bulk. Additionally, these band-bending states would offer additional scattering paths to carriers within the topologically protected surface states, decreasing the scattering time, reducing the surface state mobility and, crucially, transforming the system from a simple Dirac system to a more complex Dirac and 2D system. This will add a significant parabolic perturbation to the linear Dirac states, resulting in a modification to the effective mass and the apparent loss of the π phase [2]. It is important to note that this is not the loss of the topological protection of the surface states, merely a modification of the Dirac dispersion under an applied bias, which has been observed in previous studies of quantum transport within gated TI samples [6], [33].

## 4 Conclusions

We have investigated the electrical properties of a Bi_2_Se_3_ TI sample under the effects of an applied top gate bias in both the low frequency and THz regime. The magnetotransport appears dominated by the properties of the bulk carriers, which manifest as a smooth background in the sheet resistance and a large number of low mobility carriers contributing to the Hall conductivity. The topologically protected surface carriers contribute to SdH oscillations, visible in the second derivative of sheet resistance versus magnetic field and the THz conductivity spectrum. On application of a gate bias, we find that the density of the high mobility carriers is unaffected, whereas the carrier density of the low mobility carriers and the mobility of both carrier species are modulated by the applied electric field. Additionally, the scattering time and the mobility of the surface states are affected by the application of an electric field. We attribute these effects to the emergence of gate-induced band bending states at the top surface, which will add more scattering paths to the surface states, reducing their mobility. As Bi_2_Se_3_ is majority n-type, these states would only form in the presence of a negative gate bias, as a positive gate bias would not cause a confining potential to be formed. As such, we have shown not only that the topologically protected carriers play a key role in the THz response of these samples but also that they are extremely sensitive to the coupling between topologically trivial and non-trivial carriers due to the sensitivity of the THz radiation to the transport scattering timescale. This is of particular importance to the design of future topological plasmonic devices, as the dispersion and linewidth of the surface plasmon within TIs is dependent on the high-frequency scattering timescale, through both the conductivity of the surface states and the optical permittivity of the material [3], [34].

We note that we have not been able to modulate our Bi_2_Se_3_ through the Dirac point, as has been accomplished in several other studies of TI materials [6], [15], [16]. It is possible that to modulate the density of the surface carriers effectively, a dual gating approach is required, so that the density of the top and bottom surfaces can be tuned in tandem (as is explored in References [15] and [16]) or the dimensions of the sample need to be small so that the bulk does not screen the action of the gate from the side-channels, as in Reference [6].

Examining the THz conductivity spectrum as the sample is tuned through the Dirac point would represent a fascinating opportunity to study the interaction of the topologically protected surfaces and the residual bulk carriers that manifest within these material systems.

## Supplementary Material

Supplementary Material Details

## References

[1] Kane C. L., Mele E. J. (2005). Quantum spin hall effect in graphene. *Phys. Rev. Lett.*.

[2] Taskin A. A., Ando Y. (2011). Berry phase of nonideal Dirac fermions in topological insulators. *Phys. Rev. B*.

[3] Khanikaev A., Trevino J., Krusin-Elbaum L., Menon V., Deshko Y. (2016). Surface plasmon polaritons in topological insulator nano-films and superlattices. *Opt. Express*.

[4] Autore M. (2017). Terahertz plasmonic excitations in Bi_2_ Se_3_ topological insulator. *J. Phys.: Condens. Matter*.

[5] Stauber T., Gómez-Santos G., Brey L. (2017). Plasmonics in topological insulators: spin–charge separation, the influence of the inversion layer, and phonon–plasmon coupling. *ACS Photonics*.

[6] Steinberg H., Laloë J. B., Fatemi V., Moodera J. S., Jarillo-Herrero P. (2011). Electrically tunable surface-to-bulk coherent coupling in topological insulator thin films. *Phys. Rev. B*.

[7] Wang Y., Ginley T. P., Zhang C., Law S. (2017). Transport properties of Bi_2_(Se_1−x_Te_x_)_3_ thin films grown by molecular beam epitaxy. *J. Vac. Sci. Technol., B: Nanotechnol. Microelectron.: Mater., Process., Meas., Phenom.*.

[8] Pietro P. D. (2013). Observation of Dirac plasmons in a topological insulator. *Nat. Nanotechnol.*.

[9] Chen S. (2022). Real-space nanoimaging of thz polaritons in the topological insulator Bi_2_ Se_3_. *Nat. Commun.*.

[10] Kabir N. A. (2006). Terahertz transmission characteristics of high-mobility GaAs and InAs two-dimensional-electron-gas systems. *Appl. Phys. Lett.*.

[11] Lloyd-Hughes J., Jeon T.-I. (2012). A review of the terahertz conductivity of bulk and nano-materials. *J. Infrared, Millimeter, Terahertz Waves*.

[12] Ginley T. P., Law S. (2018). Coupled Dirac plasmons in topological insulators. *Adv. Opt. Mater.*.

[13] Luryi S. (1988). Quantum capacitance devices. *Appl. Phys. Lett.*.

[14] Zhao Y. (2014). Crossover from 3d to 2d quantum transport in Bi_2_ Se_3_/In_2_ Se_3_ superlattices. *Nano Lett.*.

[15] Chen J. (2011). Tunable surface conductivity in Bi_2_ Se_3_ revealed in diffusive electron transport. *Phys. Rev. B*.

[16] Yang F. (2015). Dual-gated topological insulator thin-film device for efficient fermi-level tuning. *ACS Nano*.

[17] Wu L. (2016). Tuning and stabilizing topological insulator Bi_2_ Se_3_ in the intrinsic regime by charge extraction with organic overlayers. *Appl. Phys. Lett.*.

[18] Mondal M. (2018). Electric field modulated topological magnetoelectric effect in bi_2_se_3_. *Phys. Rev. B*.

[19] Ireland R. M., Wu L., Salehi M., Oh S., Armitage N. P., Katz H. E. (2018). Nonvolatile solid-state charged-polymer gating of topological insulators into the topological insulating regime. *Phys. Rev. Appl.*.

[20] Knox C. S. (2024). S. S. M. at https://doi.org/10.1515/nanoph-2023-0690 for additional information about the evolution of the sample properties over the course of device properties and a summary of the Drude-Lorenz Fitting as a function of gate. *Bias*.

[21] Vaughan M. (2022). Directed delivery of terahertz frequency radiation from quantum cascade lasers within a dry 3he dilution refrigerator. *Rev. Sci. Instrum.*.

[22] Bacon D. R. (2016). Free-space terahertz radiation from a LT-GaAs-on-quartz large-area photoconductive emitter. *Opt. Express*.

[23] Kim M. H., Yan J., Suess R. J., Murphy T. E., Fuhrer M. S., Drew H. D. (2013). Photothermal response in dual-gated bilayer graphene. *Phys. Rev. Lett.*.

[24] Krewer K. L. (2018). Accurate terahertz spectroscopy of supported thin films by precise substrate thickness correction. *Opt. Lett.*.

[25] Whitney W. S. (2017). Gate-variable mid-infrared optical transitions in a (bi1-xsbx)2te3 topological insulator. *Nano Lett.*.

[26] Reed M. A., Kirk W. P., Kobiela P. S. (1986). Investigation of parallel conduction in GaAs/Al_X_Ga_1−X_As modulation-doped structures in the quantum limit. *IEEE J. Quantum Electron.*.

[27] Wieder H. H. (1974). Transport coefficients of InAs epilayers. *Appl. Phys. Lett.*.

[28] Zhang Y., Tan Y. W., Stormer H. L., Kim P. (2005). Experimental observation of the quantum hall effect and berry’s phase in graphene. *Nature*.

[29] Knox C. S. (2022). Effects of structural ordering on infrared active vibrations within Bi_2_ ( Te _(1−x)_ Se_x_ )_3_. *Phys. Rev. B*.

[30] Akrap A. (2012). Optical properties of Bi_2_Te_2_Se at ambient and high pressures. *Phys. Rev. B*.

[31] Autore M. (2015). Plasmon-phonon interactions in topological insulator microrings. *Adv. Opt. Mater.*.

[32] Nasir S., Wang Z., Mambakkam S. V., Law S. (2021). In-plane plasmon coupling in topological insulator Bi_2_ Se_3_ thin films. *Appl. Phys. Lett.*.

[33] Jauregui L. A., Pettes M. T., Rokhinson L. P., Shi L., Chen Y. P. (2015). Gate tunable relativistic mass and berry’s phase in topological insulator nanoribbon field effect devices. *Sci. Rep.*.

[34] Wu J.-S., Basov D. N., Fogler M. M. (2015). Topological insulators are tunable waveguides for hyperbolic polaritons. *Phys. Rev. B*.

[35] Knox C. S., Vaughan M. T., Fox N. R., Yagmur A., Sasaki S., Cunningham J. E., Linfield E. H., Davies A. G., Freeman J. R. (2024). Dataset Associated with Optical Conductivity of a Bi2Se3 Topological Insulator with a THz Transparent Top Gate [Dataset]. ..

