# Supplementary material for: Optical conductivity of a Bi2Se3 topological insulator with a THz transparent top gate
Source: Nanophotonics. 2024 Feb 5;13(10):1843–50. doi: 10.1515/nanoph-2023-0690 (PMC11501874; doi:10.1515/nanoph-2023-0690)
Supplement: Supplementary file 1 — Supplementary Material Details [file j_nanoph-2023-0690_suppl_001.docx]

# Supplementary Information for “Optical Conductivity of a Bi_2_Se_3_ Topological Insulator with a THz Transparent Top Gate”

## Effects of Fabrication on the Optical Spectrum

Bi_2_Se_3_ and other topological insulators have been known to degrade upon exposure to atmosphere. As such, an examination of the properties of similar Bi_2_Se_3_ samples after several fabrication steps is necessary to determine to what extent the properties of the pristine film are modified by the processing.

b)

a)

Figure S 1:Comparison of the THz conductivity of two Bi_2_Se_3_ pieces before (black) and after (red) chemical wet etching. a) Shows the real part of the conductivity spectrum, while b) shows the imaginary part.

The conductivity spectrum, as shown in Figure S1, seems only mildly effected by the chemical wet etching process. The sample was etched in a diluted Chrome etch solution (3:1 H_2_O:Ceric Ammonium Nitrate based etchant). From the THz conductivity analysis, the conductivity is comparable before and after etching ((0.096±0.003) MSm^-1^ and (0.092±0.003) MSm^-1^ respectively) however, the scattering timescale increases significantly after the etching process (0.100±0.008 ps before and 0.46±0.01 ps after). It is possible that the water used in the wet etching process intercalates between the VdW bonded layers of the Bi_2_Se_3_ crystal.

a)

b)

Figure S 2: Comparison of the THz conductivity of two Bi_2_Se_3_ pieces before (black) and after (red) deposition of the Al_2_O_3_ dielectric. a) Shows the real part of the conductivity spectrum, while b) shows the imaginary part.

The deposition of the Al_2_O_3_ dielectric has a much greater effect on the conductivity spectrum, as shown in Figure S2. However, unlike the etching process, the scattering timescale for this sample does not appear to change during the deposition process (0.12 ±0.02 ps before and after), but the conductivity is significantly different; changing from (0.067±0.001) MSm^-1^ before deposition and
(0.081±0.001) MSm^-1^ after. This is indicative of an increase in carrier density over the course of the ALD process. It is unclear whether this is due to the water precursor used within the thermal ALD process or the long period spent at an elevated temperature (100 ⁰C for 5 Hrs).

Figure S 3: Transmission through various metal thin films on encapsulated Bi_2_Se_3_.

Figure S3 shows transmission through a Bi_2_Se_3_ sample with various overlayers; Al_2_O_3_ dielectric (blue), the THz transparent Ni_0.8_Cr_0.2_ (black), Chrome (green), and Titanium (red). Chrome was found to obscure all the THz information from the Bi_2_Se_3_ and so was deemed to be opaque to THz radiation, whereas titanium appears to be translucent, but also distorts the shape of the phonon response in the Bi_2_Se_3_, and so was not pursued as a top gate electrode material. In contrast the Ni_0.8_Cr_0.2_ retains the shape of the phonon mode when compared to the encapsulated film, so was found to work well as a transparent gate electrode.

## Fitting Summary;

Here is a summary of the fitting of the real part of the conductivity of the gated Bi_2_Se_3_ shown in the main text.

Here is a summary of the imaginary part of the conductivity from the same spectra. Both parts of the conductivity spectrum were fitted simultaneously.
